# Genetics and epigenetics: paternal adolescent ethanol consumption in serotonin transporter knock-out rats and offspring sensitivity to ethanol

**DOI:** 10.1007/s00213-022-06195-5

**Published:** 2022-08-08

**Authors:** Sahir Hussain, Heidi M. D. Lesscher, Darren J. Day, Bart A. Ellenbroek

**Affiliations:** 1 School of Psychology, Victoria University of Wellington, PO Box 600, 6104 Wellington, New Zealand; 2 Department of Population Health Sciences, University of Utrecht, Yalelaan 2, 3584 CM Utrecht, the Netherlands; 3 School of Biological Sciences, Victoria University of Wellington, PO Box 600, 6104 Wellington, New Zealand

**Keywords:** Alcohol use disorder, Alcohol drinking, Serotonin transporter, Paternal drinking, Ethanol

## Abstract

**Rationale:**

Alcohol use disorder (AUD) is shown to have an overall heritability of around 50%. One of the genes associated with AUD is *SLC6A4* (solute carrier family 6 member A4) which codes for the serotonin transporter (SERT). The study looked at serotonin dysfunction on ethanol consumption in adolescents and the subsequent intergenerational effects of drinking by using a rat model: SERT^+/+^ (regular functioning), SERT^+/−^ (50% transporter reduction) and SERT^−/−^ (complete reduction).

**Objectives:**

We investigated sex and genotype differences in ethanol consumption in SERT knock-out Wistar rats (F0) followed by studying behaviour in the offspring (F1) of the male drinkers to assess effects of paternal alcohol consumption.

**Methods:**

An intermittent access two-bottle choice paradigm (IA2BC) was used to yield ethanol drinking behaviour in F0 adolescent Wistar rats. The highest drinking males were mated to alcohol-naive females and their offspring were compared with controls. Drinking behaviour (IA2BC) and ethanol-induced motor coordination effects (via rotarod) were measured in the F1s.

**Results:**

F0 drinking saw no SERT genotype differences in males. However, females consumed higher volumes of ethanol compared to males, with SERT^−/−^ females showing the highest intake. A clearer genotype effect was seen in the F1 animals, with reduction in SERT activity leading to enhanced ethanol intake in both sexes. Importantly, paternal exposure to ethanol significantly reduced the ethanol induced motor side effects in offspring, independent of sex and genotype.

**Conclusions:**

These indicate a difference in the way genetic factors may act across sexes and suggest the involvement of epigenetic mechanisms in the intergenerational effects of alcohol.

## Introduction

Alcohol is the most commonly consumed substance of abuse. In their 2021 report, the Ministry of Health states that 80% of the New Zealand general population (aged above 15 years) had used alcohol, at least once. One in five New Zealanders showed hazardous alcohol consumption, which was highest (35%) among 18–24 year olds (Ministry of Health 2021). Hazardous drinking is characterized by a compulsive pattern of irregular, high-frequency drinking (Babor et al. 2001). Moreover, hazardous drinking in adolescence is considered a risk factor for the development of alcohol dependence or alcohol use disorder (AUD) and other psychiatric disorders in adulthood (Witt 2010). Additionally, a global burden of disease study notes that there has been an estimated 50% increase in alcohol related deaths between 1990 and 2010 (Lozano et al. 2012).

While there are multiple societal factors associated with hazardous alcohol consumption, it has also been well established that alcohol abuse and alcoholism (from here on referred to as alcohol use disorders, AUD) runs in families. This strongly suggests the involvement of genetic factors, which is supported by family, twin, and adoption studies. A meta-analysis of twelve twin and five adoption studies showed an overall heritability of almost 50% (Verhulst et al. 2015), which is relatively similar to the heritability of most other mental disorders (Ellenbroek and Youn 2016). Multiple individual genetic factors have been proposed and there is every reason to assume that not a single genetic factor alone plays a decisive role in the aetiology of AUD.

Among the genes that have been associated with AUD is *SLC6A4* (solute carrier family 6 member A4) which codes for the serotonin transporter (SERT). *SLC6A4* is likely the most investigated gene in psychiatry as it encompasses several quite common polymorphisms. These include the variable number of tandem repeats (VNTR) in the promotor region known as the short and long allele version of the 5-hyroxytryptamine transporter-linked polymorphic region (5-HTTLPR) and another VNTR in intron 2, known as the Stin2 (carrying 9, 10 or 12 copies of a 17 base-pair sequence). Several human studies as well as meta-analyses have found an association between *SLC6A4* variants, alcohol use, and AUD (Feinn et al. 2005; Florez et al. 2008; Villalba et al. 2015), as well as with other substance use disorders (SUD) (Cao et al. 2013). However, research on these associations show mixed results, as studies have implicated both the short (Feinn et al. 2005, van der Zwaluw et al. 2010) and the long allele (Kweon et al. 2005) with AUD, while others have noted no association (Villalba et al. 2015) and some even suggest differences in associations based on sample population ethnicity (Cao et al. 2013) and sex (Vaht et al. 2014). A genetic role for the SERT in SUD is also supported by animal research, with research showing that a genetic reduction in SERT increases self-administration of cocaine and MDMA (Homberg et al. 2008; Oakly et al. 2013) but not heroin (Brox and Ellenbroek 2018).

So far only a few studies have investigated the genetic influence *SLC6A4* on alcohol intake in non-human subjects. A study using adult male mice found a slight reduction in alcohol intake in SERT^−/−^ when compared to their SERT^+/−^ and SERT^+/+^ counterparts (Lamb and Daws 2013). On the other hand, studies on adult rats did not reveal any influence of the SERT genotype on alcohol consumption but did show sex differences, with females consuming more alcohol per body weight than males (Klein 2015). Interestingly, adolescent male cynomolgus macaques homozygous for the short allele of the rh5-HTTLPR (an orthologue of the human 5-HTTLPR) were more sensitive to the intoxicating effects of alcohol than carriers of the long allele (Barr et al. 2003), while female adolescent carriers of the short allele of the rh5-HTTLPR s-allele exhibited higher levels of alcohol preference than l/l animals (Barr et al. 2004). It is unclear whether the differences between rodents and macaques are due to species or age differences, as the studies in rodents were done in adulthood, while the alcohol consumption in monkeys was determined during adolescence. Additionally, prior rodent SERT and ethanol studies generally use self-administration paradigms rather than home cage drinking. As an operant chamber acts as a completely different environment to the home cage, drinking behaviour is not always the same (Priddy et al. 2017). Therefore, in the present study, we wish to investigate home cage ethanol intake during adolescence in male and female rats with a genetic reduction in the SERT.

While genetic factors undoubtedly contribute to the aetiology of AUD, there is overwhelming data implicating environmental and epigenetic factors as well, such as familial alcohol consumption. Having a heavily drinking father predicts earlier onset and heavier adolescent drinking in the offspring (Vermeulen-Smit et al. 2012). One early study showed that non-alcoholic men with an alcoholic first-degree relative had a decrease in reaction intensity to ethanol compared to controls (Schuckit 1985). Physiological changes can also be seen, for example plasma cortisol levels are lower in sons of alcoholics (Schuckit 1987). More recently, it was found that individuals from families with a history of alcohol use disorder present heightened sensitivity to alcohol-induced heart rate stimulation compared to counterparts without such history (Caneto et al. 2018). People with a family history of AUD are also less sensitive to the subjective effects of alcohol compared to non-familial history AUD peers (Caneto et al. 2018). However, studies in humans do not allow to determine whether these changes in the offspring are due to imitation (Webb and Baer 1995), parental rules and norms (Van Der Vorst et al. 2006), (epi)genetic factors (reviewed in, Yohn et al. 2015), or a combination of these.

Rodent research suggests that at least some of the behavioural effects of paternal alcohol consumption may be due to biological changes such as alterations in the epigenome (Finegersh et al. 2015), as male rodents are typically removed from pregnant females before the pups are born and hence only contribute the sperm to its offspring. Finegersh and Homanics (2014) found that ethanol exposed B6 mice, when mated with ethanol naïve females, produced offspring with reduced ethanol preference in a two-bottle free choice paradigm of consumption, but higher sensitivity to the anxiolytic and motor-enhancing effects of ethanol. The results of this study were successfully replicated (Rompala et al. 2017). These effects were selective for males, as no differences were seen between ethanol- and control- female offspring (Finegersh and Homanics 2014; Rompala et al. 2017).

The current study aimed to further investigate the role of genetic variations in the SERT in the development of alcohol consumption in both male and female rats, in relation to the intergenerational effects of alcohol on the offspring, with a special focus on adolescent alcohol consumption.

## Materials and methods

### Animals

The *Slc6a4*^1Hubr^ rats used in this study have originally been described by Smits and colleagues (Smits et al. 2006) and characterized extensively elsewhere (Homberg et al. 2007; Olivier et al. 2008). In its basic form, homozygous SERT^−/−^ rats have a complete lack of *Slc6a4* mRNA and SERT protein, while heterozygous SERT^+/−^ rats have about a 50% reduction in mRNA and protein compared to wildtype SERT^+/+^ rats.

The first group (F0 generation) consisted of fifty ethanol-naive SERT Wistar rats (males *n* = 24, females *n* = 26) which were characterized for alcohol consumption in an intermittent-every-other-day two-bottle choice paradigm. Animals in this cohort were between postnatal day (PND) 35 and 37 on the first day of ethanol drinking (see below) and males (SERT^+/+^ *n* = 8, SERT^+/−^ *n* = 11, SERT^−/−^ *n* = 5) weighed between 168 and 213 g while females (SERT^+/+^ *n* = 9, SERT^+/−^ *n* = 11, SERT^−/−^ *n* = 6) weighed between 130 and 168 g at the start of the experiment. F0 animals were randomly selected from the labs stock SERT animals which come from mating SERT^+/−^ breeder animals to produce all 3 genotypes.

**Fig. 1:**
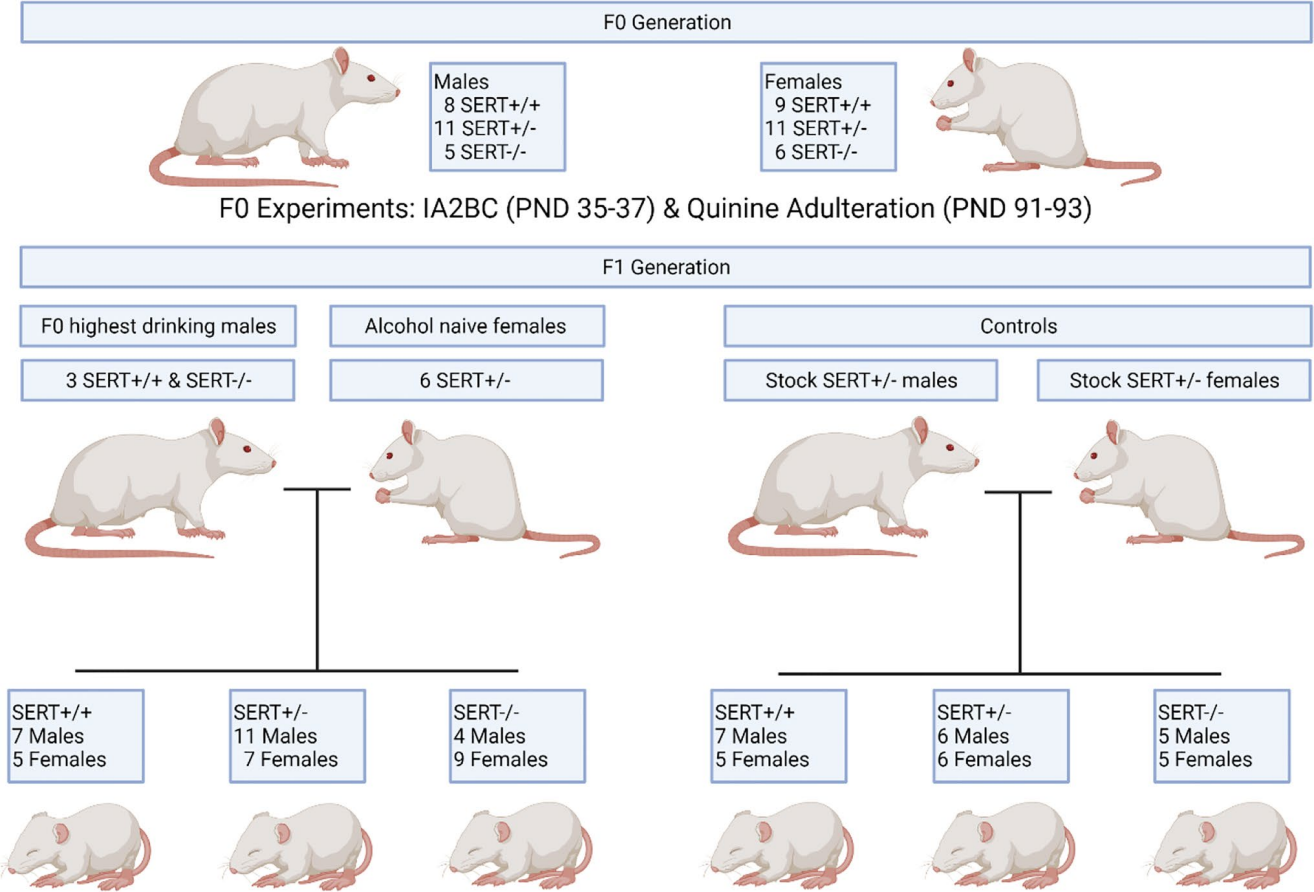
Illustration of the sample sizes, sequence and experiments performed in each generation. PND, postnatal day, IA2BC, intermittent access 2-bottle choice. Created with BioRender.com

After the alcohol consumption experiments, the 3 highest drinking SERT^+/+^ and SERT^−/−^ male rats were selected based on average g/kg ethanol intake across the consumption sessions and were kept for 4 weeks without access to ethanol before mating with 6 alcohol-naive Wistar SERT^+/−^ females (Fig. 1). This was to allow for a complete spermatogenesis cycle and to ensure no residual ethanol was present in the males (van Haaster and de Rooij 1993). The second group (F1 generation) consisted of 43 rats, 22 males (SERT^+/+^ *n* = 7, SERT^±^ *n* = 11, SERT^−/−^ *n* = 4), and 21 females (SERT^+/+^ *n* = 5, SERT^+/−^ *n* = 7, SERT^−/−^ *n* = 9) which were the progeny of the three highest drinking SERT^+/+^ and SERT^−/−^ males from F0 (drinker offspring). Additionally, 34 rats randomly selected from the lab SERT animal stock formed a control group for the F1 generation (F1 controls). The controls consisted of 18 males (SERT^+/+^ *n* = 7, SERT^+/−^ *n* = 6, SERT^−/−^ *n* = 5) and 16 females (SERT^+/+^ *n* = 5, SERT^+/−^ *n* = 6, SERT^−/−^ *n* = 5). F1 animals were PND 25–30 when starting the accelerating rotarod and PND 40–45 when starting their ethanol drinking.

All subjects used in this study were bred at Victoria University of Wellington, New Zealand, vivarium. Animals were housed with same genotype and sex littermates 3–4 per standard cage with access to food and water ad libitum in a temperature (20 °C) and humidity (60%) controlled room where they were maintained on a 12-h reverse light/dark cycle (lights off at 7 am). Animals were housed individually during testing for ethanol consumption. Animal care procedures and experimental protocols met with Victoria University ethics committee approval.

### Procedure

#### Intermittent alcohol access two-bottle choice paradigm

Rats were trained to consume ethanol (20% v/v) in their home cages using an adapted version of the intermittent alcohol access two-bottle choice paradigm (IA2BC) (Simms et al. 2008) as intermittent access has shown to induce and maintain elevated levels of voluntary ethanol consumption in lab rats which has historically been a challenge (Spoelder et al. 2015, 2017). Under this regime, rats were offered a bottle with ethanol for 7 h/day for 3 days/week (Monday, Wednesday, and Friday) for twelve sessions. On ethanol drinking days, rats were offered two plastic bottles with stainless steel, drip proof, and double ball bearing drinking spouts containing either 20% (v/v) ethanol or tap water. Bottles were presented 30 min after lights off and remained until 7 hours had passed. The ethanol bottle was replaced with a second water bottle until the next session began. After the initial 4 weeks, the rats were given access to 20% (v/v) ethanol for 24 h/day for 3 days/week for another 12 sessions. Ethanol was again presented 30 min after lights off and the bottle remained on for 24 hours after which it was replaced with water. Placement of ethanol bottles was alternated for each drinking session to control for side preferences. Ethanol and water intake were recorded by weighing the bottles before and after each session to calculate alcohol intake and preference. Bodyweight was measured weekly to calculate g/kg intake while preference was measured as a percentage of 20% (v/v) ethanol consumed to total volume of fluid intake during each session. The F0, F1, and F1 controls all underwent the IA2BC (Fig. 1).

Following 8 weeks of alcohol consumption in the F0 generation only, the three genotypes were compared for sensitivity to quinine adulteration (Lesscher et al. 2010; Spoelder et al. 2015). Quinine is a bitter compound, and its introduction was aimed to indicate inflexible drinking behaviour where the aversive taste would reduce intake. Therefore, the ethanol solution was combined with six graded concentrations (0.003, 0.01, 0.03, 0.1, 0.3, and 1 gm/l) of quinine hydrochloride dihydrate in 24-h drinking sessions during which volumes consumed were determined after 2, 7, and 24 hours. The same Monday-Wednesday-Friday paradigm was kept with quinine concentrations increasing on each ethanol presentation day, having the F0 generation drink for a total of 10 weeks (Fig. 1).

#### Ethanol-induced motor-coordination on the accelerating rotarod

Before the F1 generation and F1 controls underwent the IA2BC, they were assessed on their ethanol-induced motor-coordination, which was determined using an accelerating rotarod treadmill (Rotarod LE 8500 (76–0239), L × 50, H × 36, W × 24 cm, Panlan, Barcelona, Spain). The apparatus consists of a four-station rotating roll (60-mm diameter) mounted 10 cm above four corresponding drop levers that register the time and speed in rotations per minute (rpm) at which an animal falls from the roll. The rat must balance on the roll and adjust to increasing speeds from 4 to 40 rpm over a 5-min period. On training days, animals were habituated to the testing room for 10–15 min. Over the three training days, each animal was given three trials daily with 5–10-min inter-trial intervals. The aim of the training was to build up to 12 rpm. The last training day involved increasing the speed of the rotarod every 30 s with a minimum inclusion criterion of 15 s on the highest (12 rpm) speed being set. Forty-eight hours after the last training session, each animal received an IP injection of saline, 1.0 g/kg or 2.0 g/kg of 20% (v/v) ethanol solution. Doses were counter-balanced, and animals were given 2 days before being administered a different dose. The rats were placed on the rotarod after 10 min to allow ethanol to take effect and again after 30 min had passed, latency to fall was recorded over two trials at each of these time points. There were no exclusion criteria for any of the test conditions. Ethanol-induced ataxia scores were analysed by comparing the latency to fall at each condition between parent genotypes, animal genotypes, and sex. Accelerating rotarod testing occurred during the dark cycle with rats being counterbalanced to control for any time-of-day effects.

### Solutions

Ethanol 20% (v/v) was made through dilution of 99.8% ethanol solution (Purescience Ltd, Porirua New Zealand) with distilled water. Quinine solutions were formed by adding quinine hydrochloride dihydrate (Sigma- Aldrich Co., St. Louis, Mo, USA) in the required amounts to the already made 20% ethanol solutions.

### Data analysis

Data was analysed using the computer software IBM Statistical Package for the Social Sciences (SPSS). A generalised linear mixed model analysis with an autoregressive covariance structure was used for ethanol consumption. This model was chosen to accommodate for days during which data of a subject was lost due to leaks in the drinking bottles (Wang and Goonewardene 2004). Across the 3 cohorts, there were 8 instances of bottle leaks causing those animals to not have a drinking score on the day of the leak. Day 16 of ethanol drinking was not included for the F1 generation analyses due to an unexplained spike in ethanol consumption of the F1 controls (Appendix A).

Independent variables used were three levels of genotype (SERT^+/+^, SERT^+/−^, and SERT^−/−^) and two levels of sex (females and males) as between subject and days as within subject variables. Ethanol consumed in grammes per kilogramme (g/kg) was the dependent variable. Average ethanol preference for the 7-h and 24-h sessions, quinine adulteration across 6 doses, and ethanol-induced motor-coordination across 3 injections were measured using mixed factorial ANOVAs (analysis of variance), as no data points were missing. Animal weights were used as co-variates for the rotarod analyses.

## Results

### Ethanol consumption

#### F0 voluntary ethanol consumption and preference

**Fig. 2:**
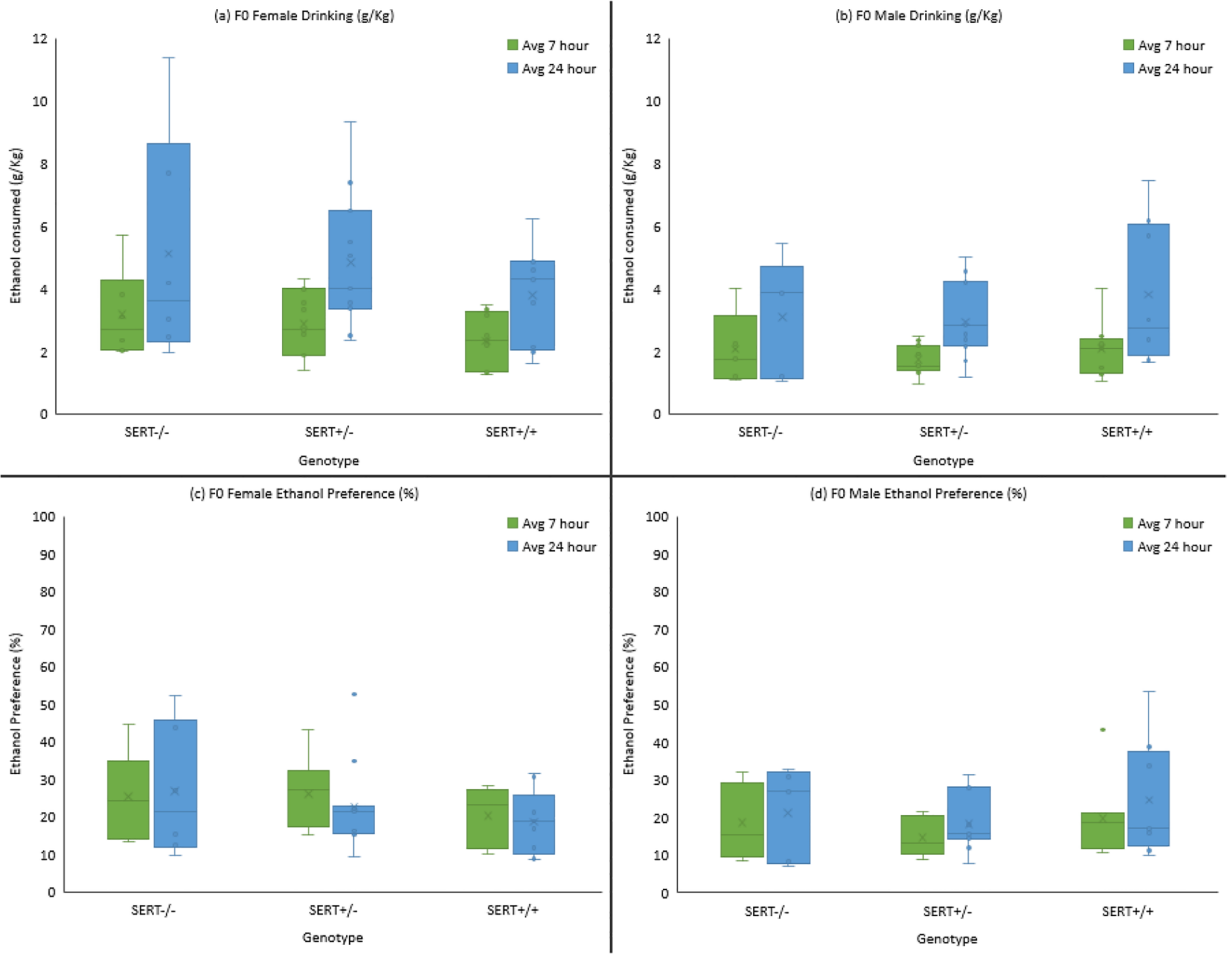
Average ethanol consumption and ethanol preference F0 generation across the 7-h and 24-h drinking sessions. SERT^−/−^ females showed the highest voluntary consumption followed by SERT^+/−^ and SERT^+/+^ (**a**) while no such pattern was observed in males (**b**). There were no differences in preference of ethanol **c** and **d** between the genotypes or across sexes

In the intermittent alcohol access paradigm, alcohol consumption was assessed over twelve 7-h sessions followed by twelve 24-h sessions (Fig. 2). Drinking significantly increased over time (*F* (23, 252.87) = 11.50, *p* < 0.001) with a significant difference between the 7-h and 24-h sessions (*p* < 0.001). The general linear mixed model’s analysis revealed a significant main effect of sex (*F* (1, 89.15) = 13.19, *p* < 0.001), with females consistently drinking more ethanol than males. While there was no main effect of genotype (*F* (2, 89.15) = 0.46, *p* = 0.636). As the sex × genotype interaction was near significance (*F* (2, 89.15) = 3.00, *p* = 0.054), further analysis of the interaction was assessed which showed that in females, SERT^−/−^ rats consumed significantly more ethanol than SERT^+/+^ (*p* = 0.037), while no significant genotype differences were found among the males (Fig. 2).

A 2 (sex) × 3 (genotype) × 2 (drinking session) mixed factorial ANOVA showed no main effects of sex, genotype, or drinking session (*p* > 0.05). There was however a significant sex x drinking session interaction (*F* (1, 44) = 6.65, *p* < 0.013). Females (*M* = 23.96, *SD* = 9.44) showed a greater preference for ethanol in the 7-h sessions than males (*M* = 17.18, *SD* = 8.17) but this difference was no longer present in the 24-h sessions with females (*M* = 22.27, *SD* = 12.28) and males (*M* = 21.03, *SD* = 11.56) having similar preference scores (Fig. 2).

#### F0 compulsive ethanol seeking during quinine adulteration

**Fig. 3:**
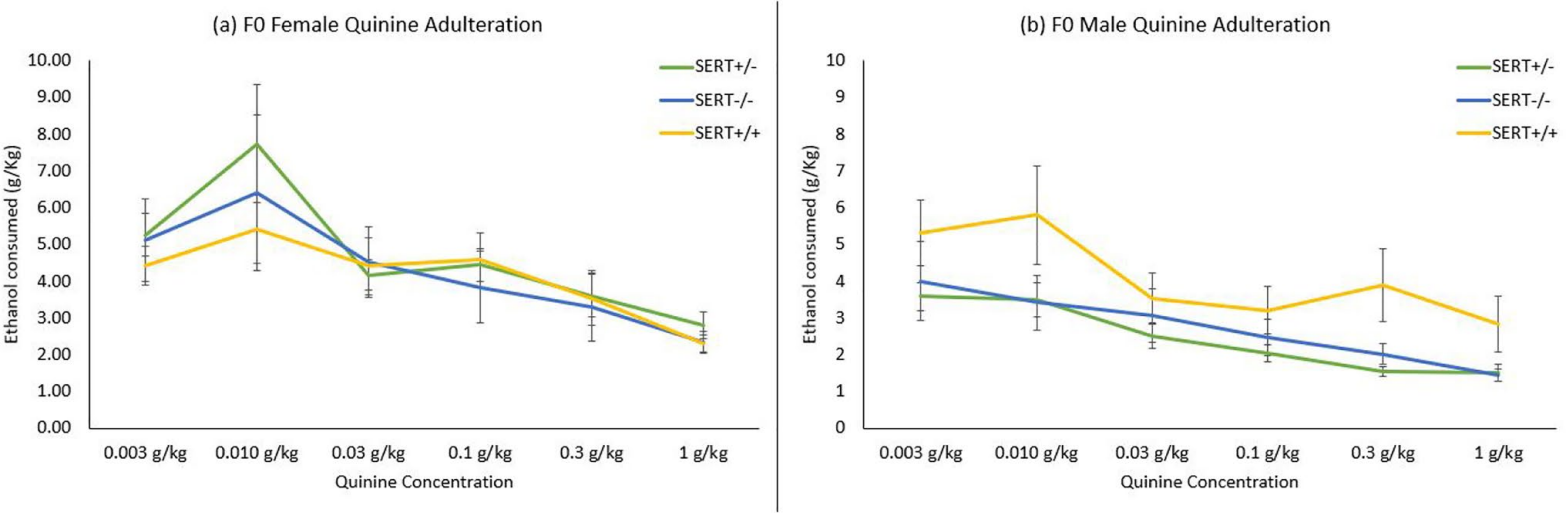
Ethanol consumption reduced with increase in dose. There was no statistically significant difference in compulsive ethanol intake between genotypes. Females (**a**) still consumed more per bodyweight than males (**b**). Bars denote standard error

Compulsive ethanol seeking was investigated over six doses of quinine being dissolved in increasing order over the same number of sessions. A 2 (sex) × 3 (genotype) × 6 (dose) mixed factorial ANOVA revealed a significant effect of dose (*F* (1, 40) = 71.68, *p* < 0.001), showing a reduction in consumption as the concentration of quinine increased (Fig. 3). Sex differences were observed (*F* (1, 40) = 4.62, *p* = 0.038) with again females consuming higher quantities of ethanol than males. However, there was no significant interaction between genotype and dose, indicating that the quinine-induced reduction in consumption was similar across genotypes (Fig. 3).

#### F1 and F1 controls voluntary ethanol consumption

**Fig. 4:**
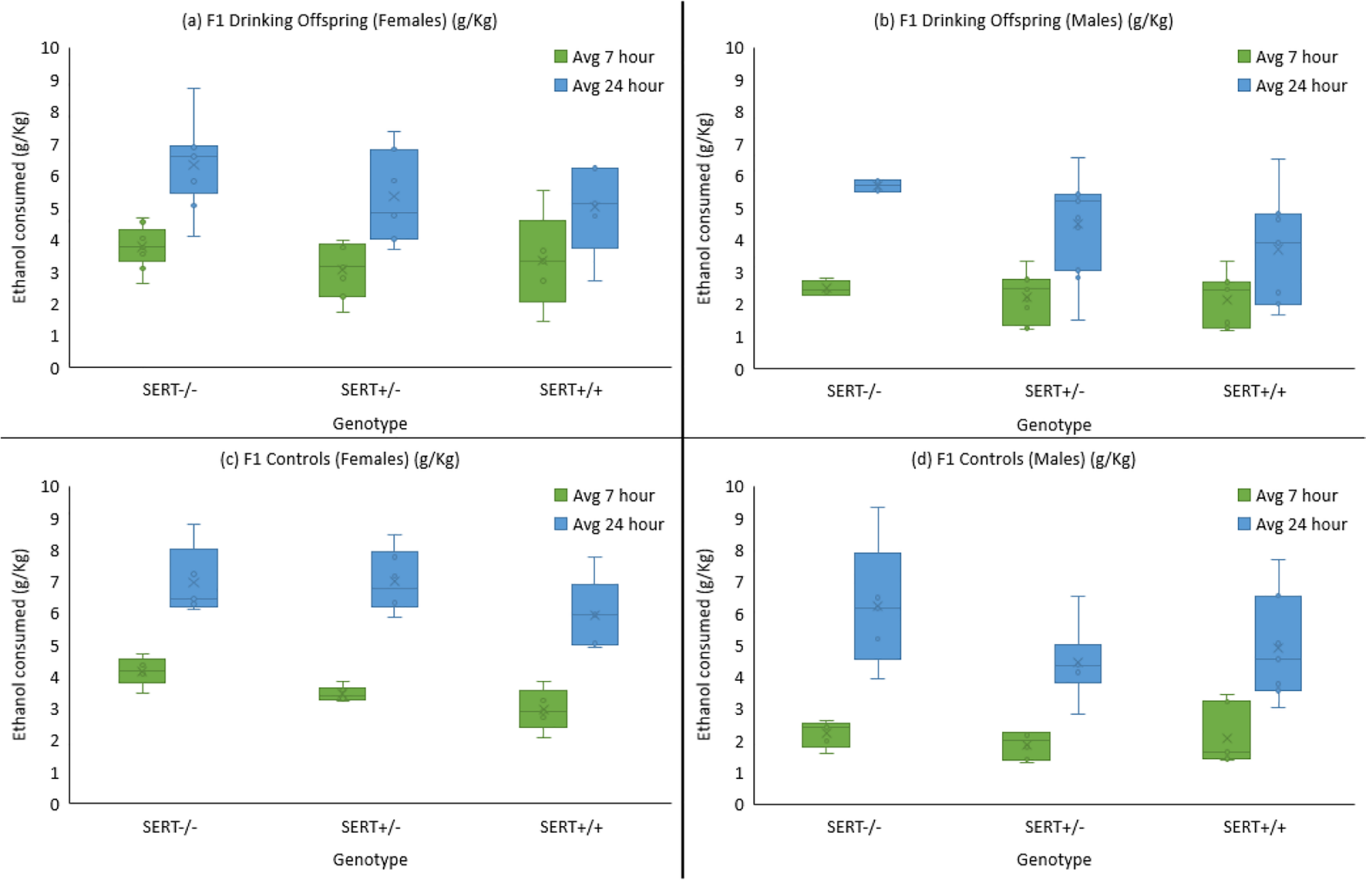
Average ethanol consumption F1 drinking offspring and controls. There was a significant main effect of genotype. SERT^−/−^ rats consumed the highest levels of alcohol of all groups. Females (**a** and **c**) always consumed significantly more alcohol than males (**b** and **d**)

A linear mixed model analysis of the F1 generation looking at the 2 (parent) × 2 (sex) × 3 (genotype) × 23 (days) again showed a significant effect of days (*F* (22, 374.241) = 26.68, *p* < 0.001), indicating an increase in alcohol consumption over the 7-to-24-h sessions (Fig. 4). In addition, there were main effects of sex (*F* (1, 147.76) = 56.65, *p* < 0.001) with females again consuming higher amounts of ethanol, and genotype (*F* (1, 147.76) = 11.82, *p* < 0.001) where the SERT^−/−^ animals showed greater drinking in both the drinker offspring and controls, which is apparent from a lack of an overall effect of parent (*F* (1, 147.76) = 0.67, *p* = 0.414). Likewise, there were no two- or three-way interactions between sex, genotype, and parent.

#### F1 and F1 controls ethanol preference

**Fig. 5:**
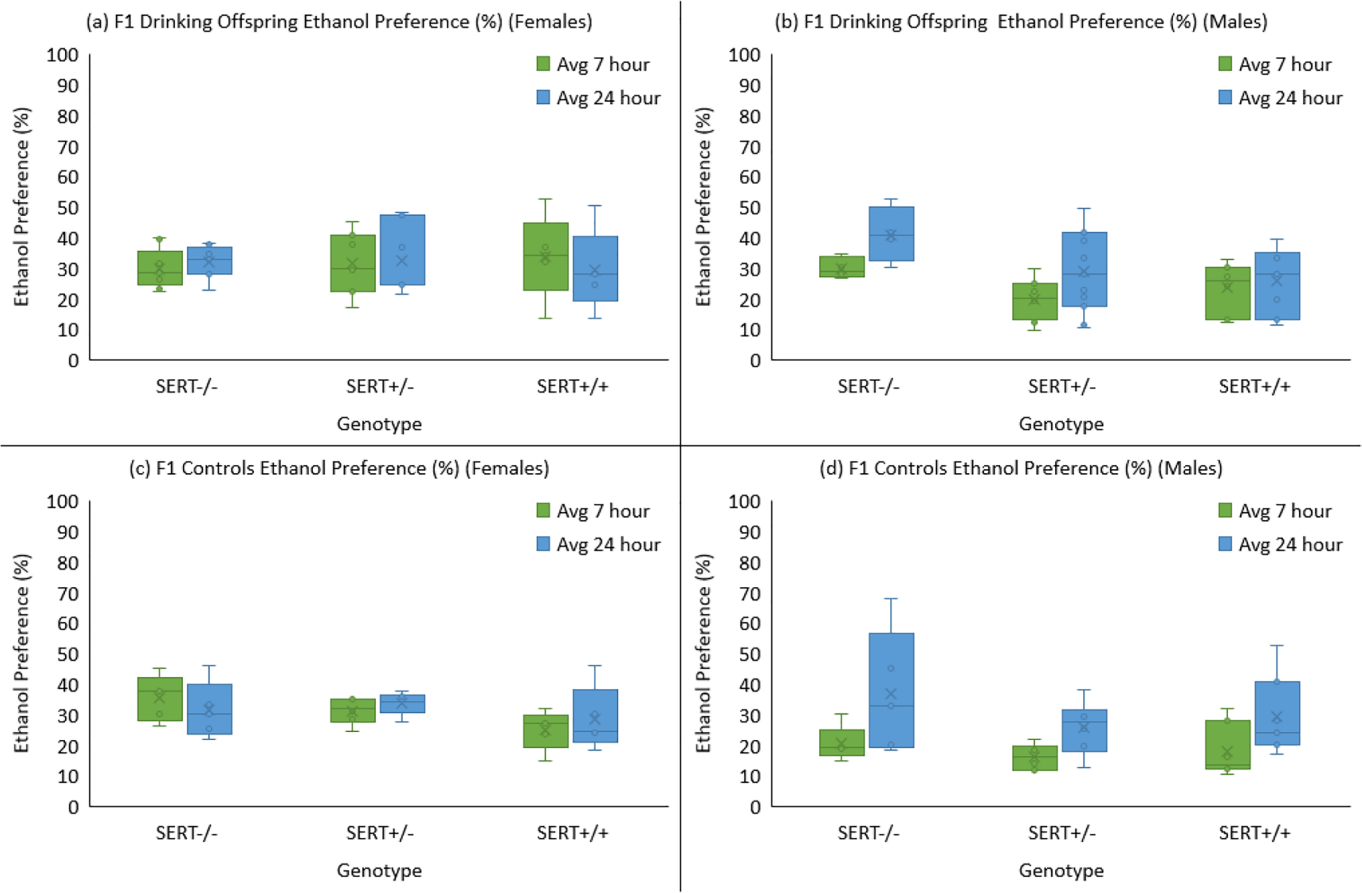
Average ethanol preference for F1 generation and matched F1 controls across the 7-h and 24-h drinking sessions. A significant effect of sex was seen with females (**a** and **c**) having greater ethanol preference than males (**b** and **d**), but this difference was not seen in the 24-h session

A 2 (parent) × 2 (sex) × 3 (genotype) × 2 (session) mixed factorial ANOVA again showed no differences between the offspring of drinkers and controls (Fig. 5). There was a significant reduction in ethanol drinking preference from the 7- to the 24-h sessions (*F* (1, 65) = 25.14, *p* < 0.001). A main effect for sex was seen with females showing a greater preference to ethanol (*F* (1, 65) = 5.68, *p* = 0.020) and there was also a sex × session interaction where the difference in preference between males and females only being seen during the 7-h sessions (*F* (1, 65) = 24.71, *p* < 0.001). Genotypes did not show a significant difference (*F* (1, 65) = 2.56, *p* = 0.085). In preference, however, they did show a trend where SERT^−/−^ showed greater preference to ethanol than SERT^+/−^ and SERT^+/+^ (Fig. 5).

#### Voluntary ethanol consumption and ethanol preference, comparison of F0 and F1 controls

While voluntary ethanol consumption was investigated in both generations of rats during adolescence, the rats in the F1 generation and the controls were first exposed to ethanol injections and subjected to the rotarod test before the intermittent-access two-bottle paradigm. To investigate whether this pre-exposure affected the voluntary ethanol intake, we compared the F0 generation with the F1 controls. Using a 2 (cohort) × 2 (sex) × 3 (genotype) × 23 (days) mixed ANOVA, we again found a significant effect of day (*F* (23, 382.29) = 58.60, *p* < 0.001), with alcohol intake increasing over time. In addition, we found a significant effect of sex (*F* (1, 66) = 16.66, *p* < 0.001), with females again drinking more than males and, most importantly, of cohort (*F* (1, 66) = 7.44, *p* < 0.001) with the animals pre-exposed to alcohol injections drinking significantly more than the F0 non-pre-exposed generation but no effect of genotype. There were no significant two- or three-way interactions between cohort, sex, and genotype.

In an assessment of preferences using a 2 (cohort) × 2 (sex) × 3 (genotype) × 2 (session) mixed ANOVA, we see a significant effect of cohort. The F1 controls showed a significantly greater preference to ethanol (*F* (1, 72) = 8.50, *p* = 0.005). We see a main effect of session (*F* (1, 72) = 21.77, *p* < 0.001) and a session × cohort interaction (*F* (1, 72) = 10.07, *p* = 0.002) that show no differences in preference in the 7-h drinking but the F1 controls had a greater preference for ethanol in the 24-h sessions. Again, we see a difference of sex with females showing greater preference to males (*F* (1, 72) = 5.68, *p* = 0.020) and a session × sex interaction where preferences are higher for females in the 7-h sessions but this goes away in the 24-h sessions (*F* (1, 72) = 25.53, *p* < 0.001). Lastly, a 3-way interaction of session × sex × cohort was seen as F1 control males showed the largest change in ethanol preference between sessions while the changes were a lot smaller for the other groups (*F* (1, 72) = 4.32, *p* = 0.041). There were no differences in genotype.

### Ethanol-induced effects on motor coordination in F1s

**Fig. 6:**
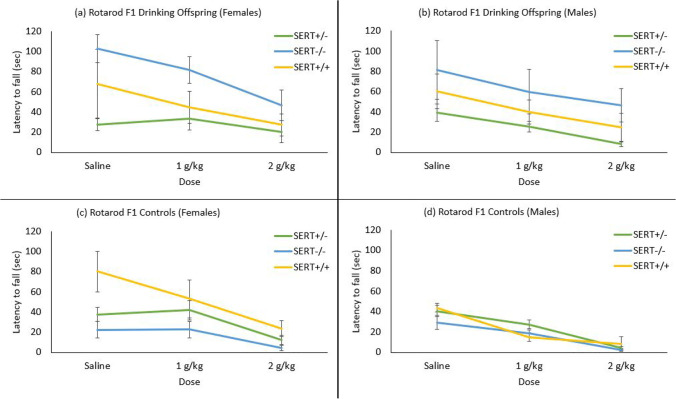
Motor coordination measured by latency to stay on a rotarod after a dose of saline, 1 g/kg ethanol, or 2 g/kg ethanol intraperitoneal injection. Animals with drinking fathers performed better on the task under the influence of ethanol. Female animals performed better than males. Bars denote standard error

Motor coordination was measured as the latency to fall from an accelerating rotarod treadmill (Fig. 6). The data were analysed with a 2 (parent) × 2 (sex) × 3 (genotype) × 3(dose) mixed model ANOVA (with dose as within subject variable and animal weights as a covariate) and revealed a significant main effect of dose (*F* (2, 154) = 4.65, *p* = 0.011) with time spent on rotarod decreasing with increase of dose. No significant sex differences were observed (*F* (1, 77) = 0.09, *p* = 0.760); however, weights showed a significant effect (*F* (1, 77) = 9.61, *p* = 0.003), which may explain the greater time spent on the rotarod seen in females over males. There was a significant genotype effect (*F* (2, 77) = 4.17, *p* = 0.019) with SERT^+/−^ performing worse than both the SERT^−/−^ and SERT^+/+^.

Importantly, the parental drinking history strongly affected motor coordination (*F* (1, 77) = 22.37, *p* < 0.001) with both male and female F1 offspring of drinking fathers showing longer latency to falling than offspring of alcohol-naive animals. As Fig. 6 indicates, while the offspring of alcohol-naive fathers showed a clear dose-dependent reduction in latency to fall, this effect was attenuated in the offspring of alcohol drinking fathers. There was also a significant parent × genotype interaction (*F* (2, 77) = 10.50, *p* < 0.001) with SERT^−/−^ animals of drinking parents showing the highest latency to fall.

## Discussion

### General

The results of the current study show that throughout the course of the 8-week exposure, rats showed an increase in ethanol consumption, an effect consistently seen in all three cohorts (the F0 generation, the F1 offspring, and the F1 controls). Quinine adulteration in the F0 reduced but did not stop ethanol consumption behaviour suggesting the rats had developed a resistance to significant reductions in drinking. In relation to the major aims, the study showed that the effects of ethanol differed between sexes, and parental drinking history affected ethanol induced motor coordination deficits, but not voluntary alcohol intake. Lastly, a trend indicating a sex × genotype interaction in alcohol consumption was seen in the F0 generation with SERT^−/−^ females consumed the highest amounts. SERT^−/−^ rats were also the highest drinkers in the F1 generation.

### Males and female rats react differently to ethanol

One of the most consistent findings from the present study was a sex difference in the sensitivity to alcohol. Both in the F0 and both F1 cohorts, females consistently consumed more ethanol per bodyweight than males but were less sensitive to the motor disruptive effects of ethanol. Additionally, females showed greater preference to ethanol in the F1 and F1 controls but not in the F0s. It is important to note that this difference was seen in the 7-h drinking sessions but not in the 24-h ones. These findings are in mostly line with previous research showing sex differences in drug use, particularly stimulant drugs (Carroll and Anker 2010). Females have higher alcohol preference ratios (alcohol/total fluid) in free choice paradigms (Lancaster and Spiegel 1992; Priddy et al. 2017; Pirino et al. 2022), wider distribution of alcohol consumption across the day (Juárez and Tomasi 1999), and higher intake of alcohol per body weight (Lancaster et al. 1996; Klein 2015). Other studies show that female mice and rats acquire ethanol self-administration faster (Melón et al. 2013; Moore and Lynch 2015), show less withdrawal symptoms (Varlinskaya and Spear 2004), and have a lower response to pharmacological treatments for AUD (Moore and Lynch 2015) compared to males. However, studies have reported greater ethanol consumption in adolescent males (Vetter-O'Hagen et al. 2009) or have found no sex differences in either adolescent (Lancaster et al. 1996) or adult (Quadir et al. 2022) rats. However, Quadir et al. (2022) did find sex-dependent interactions of affective states with ethanol intake and how the two may influence each other. The enhanced rewarding effects in females are also apparent in conditioned place preference studies, where adult females show a clear preference for ethanol, while males do not (Torres et al. 2013). Recently, while these studies differ in housing conditions, concentration of ethanol used, test parameters, and other possible environmental conditions, they all indicate sex differences to drug response.

A sex difference was also observed in the quinine adulteration experiment, though there was no interaction with the quinine dose, suggesting no differences in the urge to drink ethanol between the sexes. There were no differences in variance across drinking days between males and females which backs up past research (Vetter-O'Hagen et al. 2009). In the rotarod study, females on average performed better than males, although this effect was not significant when animal weights were added as a covariate. However, previous research looking at ethanol-induced motor impairment supports the idea of significant differences in sensitivity with females requiring higher doses to impair performances on negative geotaxis tasks (Ramirez and Spear 2010). While females do generally perform better than male animals on the rotarod task and other tests of motor coordination and grip strength (Hernandez et al. 2020), the reduced decline in average performance indicates sensitivity differences between the sexes. One reason for this dimorphism could be the differences in alcohol metabolism between the sexes with studies showing lower blood alcohol levels for the same ethanol dose injection (Mankes et al. 1991) and similar blood ethanol concentrations for greater ethanol intake (Pirino et al. 2022) which may be explained by the higher hepatic alcohol dehydrogenase activity in females compared to male rats (Quintanilla et al. 2007). However, it is currently unclear whether the enhanced drinking in females is related to the reduced side effect potential in these animals and why we see no differences in alcohol preference in the 24-h drinking sessions.

It is unclear from our data and the literature why females consume more ethanol or show different preference patterns across sessions, since increases in intake and preference can be due to both increases as well as decreases in sensitivity to the reinforcing effects of drugs of abuse (Volkow et al. 2010; Wise and Koob 2013). Oestrogen and other endocrine hormones are thought to play a role in the differences observed. The influence of hormones on the rewarding effects of ethanol is seen through ovariectomised females showing lower levels of baseline ethanol intake compared to intact females (Ford et al. 2002) and this effect being reversed with estradiol replacement (Ford et al. 2004). Although, Priddy et al. (2017) found no effect of the rat estrous cycle in drinking under intermittent access paradigms. Studies in SERT animals have shown sexually dimorphic effects due to reduction of SERT expression. Decreased SERT expression in SERT knock-out mice was associated with region specific reduced receptor expression with greater reduction in females (Li et al. 2000, 2003), while genotype-related opposing trends in mitochondrial copy number and expression of the complex I subunit mt-Nd1 expression is seen in male and female SERT Wistar rats (Thorne et al. 2022a). Whether these findings are related to the sex biases seen in addiction behaviours remains to be investigated.

### Paternal ethanol exposure reduced ethanol sensitivity in the offspring

The second major finding of the present study is that paternal exposure to ethanol significantly affected the sensitivity to ethanol in the F1 progeny. In the rotarod, we found a significant parental history × dose interaction with the offspring of drinker rats being significantly less sensitive to the motor disruptive effects than the offspring of alcohol-naive animals. Interestingly, this effect was independent of the genotype of the offspring as evidenced by a non-significant three-way interaction between parental history, dose, and genotype. As mentioned earlier, previous research performed in mice showed male offspring of ethanol drinking sires were able to perform better on the accelerating rotarod task than controls after ethanol administration (Finegersh and Homanics 2014; Rompala et al. 2017). The reduced sensitivity in motor coordination in this study is seen in both male and female offspring and fits in with research done in rodents showing male offspring of alcohol drinking fathers having a decreased preference to the rewarding effects of ethanol at high doses in conditioned place aversion and context induced relapse tasks (Ceccanti et al. 2016; Campbell et al. 2018). This is in line with work in humans suggesting that a reduced sensitivity to alcohol in sons of drinkers contributes to the enhanced vulnerability to alcoholism (Schuckit 1988). However, previous rodent research on alcohol consumption reports mixed results in offspring drinking (Finegersh and Homanics 2014; Rompala et al. 2017; Campbell et al. 2018). Our results did not reveal a reduction in alcohol consumption based on the parental ethanol drinking history, although the above-mentioned caveat (i.e., that the F1 offspring had been pre-exposed to ethanol injections) needs to be considered.

While the current study did not specifically address the underlying mechanisms, it is important to realize that the males of the F0 were ethanol-free at the time of mating and were removed from the cage before the litters were born. This strongly suggests that epigenetic alterations are important for the paternal effects on the F1 generation. Epigenetics results in altered gene expression through modification of either DNA, the surrounding histones, or changes in the expression of non-coding RNAs. One of the most studied epigenetic processes is DNA methylation, typically involving the addition of a methyl group to a cystine residue, a process mediated by DNA methyltransferases (DNMTs) and generally leading to a reduction in transcription. Ethanol inhibits DNMTs (Zhang et al. 2013) and while sperm cells are hypomethylated compared to oocytes, some genes are notably exempt. Studies report imprinted genes such as H19-IGF2 showing altered methylation due to ethanol (Ouko et al. 2009; Liang et al. 2014), and paternal alcohol exposure appears to affect brain derived neurotrophic factor (*Bdnf*) DNA methylation in offspring (Nieto et al. 2022). Given the parental differences were in ethanol induced motor coordination but not drinking behaviour, there could be brain region specific epigenetic changes which need to be understood.

### Genetically reduced SERT activity affects ethanol sensitivity

One of the main objectives of the present research was to investigate whether a genetic reduction in SERT activity affected sensitivity to ethanol. While we did not find a significant genotype effect in the F0 generation, an almost significant genotype × sex difference was found (*p* = 0.054). In addition, there was a significant genotype effect in the F1 generation, which was not dependent on parental drinking history, nor on sex. Rats with a genetic deletion of the SERT (SERT^−/−^) consumed significantly larger amounts of ethanol than wildtype SERT^+/+^ rats. Drinking preferences in F1 also showed a trend of SERT^−/−^ rats having higher ethanol preference compared to the other two genotypes. It is currently unclear why this effect was limited to females in the F0 generation but seen in both sexes in the F1. One possibility for this could just be the small sample size of SERT^−/−^ males in the F0 which can affect power and thus the lack of difference must be looked at with caution. Another major difference between the two generations (apart from the paternal drinking history) is that the F1 animals were tested in the rotarod set-up prior to the voluntary drinking paradigm and thus had been exposed to two injections of ethanol (1 and 2 g/kg). While the precise neurobiological effects of these priming injections need to be investigated, the pre-exposure significantly increased the ethanol intake and ethanol preference as evidenced by the significant cohort effects when comparing F0 with the non-drinker F1 offspring.

These data confirm and extend our knowledge of the influence of genetic alterations in SERT activity in self-administration. Previous research from our group and others has shown that a genetic reduction in SERT activity enhances cocaine (Homberg et al. 2008; Verheij et al. 2014) and MDMA (Oakly et al. 2013) but not heroin (Brox and Ellenbroek 2018) self-administration. However, it is important to note that all these previous studies were performed in male rats only. A recent review highlights the heavy sex bias in SERT research which has worsened since the turn of the millennium (Thorne et al. 2022b). Therefore, this study provides further justification to include females in order to get a full picture of the role of the SERT in drug addiction, particularly given the variability in human studies described in the introduction.

It is important to note that in our present study, we did not find a significant genotype effect in the quinine adulteration experiments, suggesting that while the SERT plays a role in the reinforcing effect of ethanol, it may be less important for compulsive drinking behaviour. There is abundant evidence to implicate the serotonin in the neurobiology of drugs of abuse (review Müller and Homberg 2015) although its precise role is complex, particularly as pharmacological, and genetic alterations in 5-HT neurotransmission can lead to opposite effects. Bellia et al. (2020) pre-treated animals with the 5-HT depleting agent p-chloro-phenylalanine (PCPA) and found that PCPA treatment significantly reduced ethanol intake. Extrapolating this, our study uses a SERT haploinsufficient model which allows for subtler variations between groups and provides a strong framework for assessing the effect of altered serotonin levels. Together, results here suggest that the increase in extracellular 5-HT levels due to the genetic reduction of SERT during adolescence may be related to the increased intake of ethanol.

## Conclusions

In the present study, we found differences in drinking behaviour between male and female rats. A genetic reduction in SERT activity does appear to influence ethanol intake, particularly in the F1s, but does not affect ethanol’s motor side effects. Importantly, we found that paternal exposure to ethanol significantly reduces the ethanol induced motor side effects in the F1 offspring, an effect which is independent of the genotype and sex. The results suggest the involvement of epigenetic mechanisms in the intergenerational effects of ethanol. Subsequent research, however, is necessary to identify the nature of these mechanisms.

## Figures and Tables

**Fig. 7:**
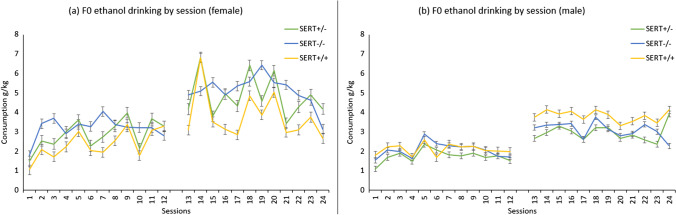
Ethanol consumption of F0. The g/kg intake increased from 7-hour sessions (days 1–12) to 24-hour sessions (days 13–24) in both females (a) and males (b). Error bars denote SEM

**Fig. 8:**
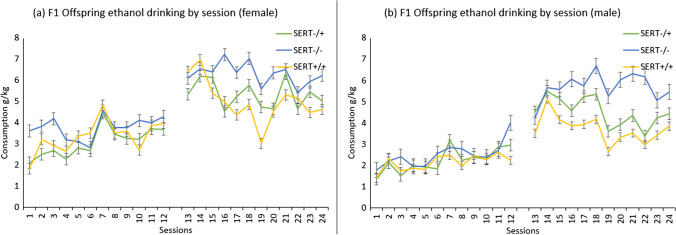
Ethanol consumption of F1 offspring. The g/kg intake g/kg increased from 7-hour sessions (days 1–12) to 24-hour sessions (days 13–24) in both females (a) and males (b). Error bars denote SEM

**Fig. 9:**
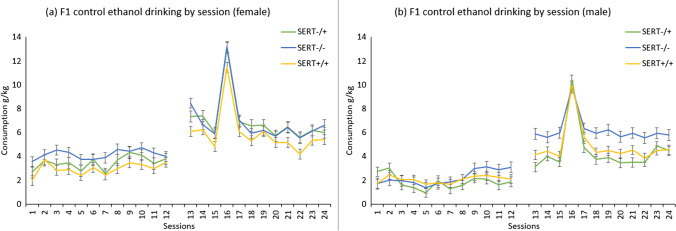
Ethanol consumption of controls for the F1 generation. The g/kg increased from 7-hour sessions (days 1–12) to 24-hour sessions (days 13–24) in both females (a) and males (b). Error bars denote SEM

## Appendices

Per day drinking data of each of the cohorts.
